# Immune biomarker evaluation of sequential tyrosine kinase inhibitor and nivolumab monotherapies in renal cell carcinoma: the phase I TRIBE trial

**DOI:** 10.1016/j.iotech.2024.100712

**Published:** 2024-03-18

**Authors:** K.S. Shohdy, M. Pillai, K.S. Abbas, J. Allison, T. Waddell, E. Darlington, S. Mohammad, S. Hood, S. Atkinson, K. Simpson, D. Morgan, P. Nathan, E. Kilgour, C. Dive, F. Thistlethwaite

**Affiliations:** 1 The Christie NHS Foundation Trust, Manchester, UK; 2 Division of Cancer Sciences, The University of Manchester, Manchester, UK; 3 Faculty of Medicine, Alexandria University, Alexandria, Egypt; 4 Cancer Biomarker Centre, Cancer Research UK Manchester Institute, Manchester, UK; 5 Mount Vernon Cancer Centre - East and North Herts NHS Trust, Northwood, Middlesex, UK

**Keywords:** predictive biomarkers, immune checkpoint blockade, metastatic renal cell carcinoma, nivolumab, progression-free survival, clinical benefit rate

## Abstract

**Background:**

Predictive biomarkers for immune checkpoint blockade in the second-line treatment of metastatic renal cell carcinoma (mRCC) are lacking.

**Materials and methods:**

Patients with histologically confirmed RCC who started nivolumab after at least 4 months of tyrosine kinase inhibitors (TKIs) were recruited for this study. Serial tissue and blood samples were collected for immune biomarker evaluation. The primary endpoint was to determine the association of specific T-cell subsets with clinical outcomes tested using Wilcoxon rank sum for clinical benefit rate (CBR) and log-rank test for progression-free survival (PFS).

**Results:**

Twenty patients were included in this trial with a median age of 64 years and followed-up for a median of 12 months. The median PFS for patients who received TKI was 13.8 months, while for those subsequently treated with nivolumab following TKI therapy, the median PFS was 2.6 months. CBR of nivolumab was 20% with two partial responses. Functionally active programmed cell death protein 1+ CD4+ T cells were enriched in non-responders (*q* = 0.003) and associated with worse PFS on nivolumab (*P* = 0.04). Responders showed a significant reduction in the effector CD4+T-cell (T_EF_) fraction compared to non-responders at 3 months on nivolumab (0.40 versus 0.80, *P* = 0.0005). CD127+CD4+ T cells were enriched in patients who developed immune-related adverse effects (*q* = 0.003). Using in-house validated multiplex immunohistochemistry for six markers, we measured tumour-associated immune cell densities in tissue samples**.** Responders to nivolumab showed a significantly higher mean of immune cell densities in tissue samples compared to non-responders (346 versus 87 cells/mm^2^, *P* = 0.04).

**Conclusions:**

In this small study, analysis of tissue-based and peripheral blood immune cell subsets predicted clinical outcomes of nivolumab. Further studies are warranted with larger populations to validate these observations.

## Highlights

• Significant reduction was found in CD4+T_EF_ cell fraction in responders at 3 months on nivolumab.
• CD127+CD4+ T cells enriched in patients with immune-related adverse effects to nivolumab.
• Higher mean immune cell densities in tissue samples correlated with responders to nivolumab.
• The study suggests the potential of immune cell subsets in predicting clinical outcomes.

## Introduction

Metastatic renal cell carcinoma (mRCC) is a fatal malignancy with a recent improvement in overall survival (OS), reaching 2-3 years after diagnosis.^1^ Several life-extending drugs have been developed to control the disease, but treatment algorithms to determine the optimal sequencing of available options are not yet defined. In the first-line (1L) settings, The American Society of Clinical Oncology (ASCO) guidelines recommend that patients falling into the intermediate or poor-risk categories should undergo assessment for combination therapy options, which may involve the use of two immune checkpoint inhibitors, such as nivolumab and ipilimumab, or an immune checkpoint inhibitor in conjunction with a vascular endothelial growth factor (VEGF) receptor inhibitor.^2^

The checkpoint inhibitor nivolumab is one of the most commonly used second-line (2L) treatments when 1L single-agent VEGF inhibition using a tyrosine kinase inhibitor (TKI) is no longer effective.^3^, ^4^, ^5^, ^6^ However, in the pivotal trial (CheckMate 025), the objective response rate to nivolumab in this setting was only 25%.^7^ Multiple trials were recently published on the promising role of nivolumab in the management of mRCC.^7^, ^8^, ^9^

Currently, there are no predictive biomarkers to identify patients with mRCC who will respond to nivolumab, nor are there data on the effect of 1L TKIs to influence the likelihood of response to 2L immunotherapy. Evaluating and correlating immune and molecular characteristics from blood and tumour may identify candidate-predictive biomarkers of immunotherapy response. Differences according to a particular 1L TKI may also be identified; these variances could influence tolerance and response to 2L immunotherapy.

The current study was designed to investigate peripheral blood and tumour tissue-based immune biomarkers in a prospective cohort of patients with mRCC receiving sequential 1L anti-VEGF TKI and 2L nivolumab. During the period when TRIBE was open, combination immunotherapy (ipilimumab + nivolumab) was approved for 1L use in this patient population.^10^ This change in the treatment landscape negatively impacted recruitment to the TRIBE study, resulting in the study closing early before the completion of recruitment. Here we report on the available data from the cohort recruited before trial discontinuation and exploratory biomarker analyses.

## Materials and methods

### Trial design

The study aimed to recruit a prospective cohort of 50 patients with mRCC who had progressed on TKIs and plan to commence nivolumab. Key inclusion criteria were histologically confirmed RCC, with at least one measurable lesion by Response Evaluation Criteria in Solid Tumours version 1.1, clear cell or mixed histology and an Eastern Cooperative Oncology Group (ECOG) performance status 0-1. Patients should have received one line of anti-VEGF TKI therapy with either pazopanib or sunitinib for at least 4 months. Patients were required to demonstrate adequate hematologic, hepatic and renal function before enrolment. Imaging was conducted before commencing nivolumab treatment, with a follow-up scheduled at 3 months. Blood was collected at time point 1 [cycle1 day 1 (C1D1) nivolumab] and time point 2 [after 12 weeks (±7 days)]. Treatment-naïve tumour tissue sampling was conducted for a subset of patients (Supplementary Figure S1, available at https://doi.org/10.1016/j.iotech.2024.100712)**.** The primary objective was to evaluate immunological signatures by estimating mean immune cell percentages as candidate predictors for clinical outcomes in patients with mRCC following disease progression on the 1L TKI therapy with either pazopanib or sunitinib. Secondary objectives included determining the association of immunological profiles at C1D1 with tolerance, progression-free survival (PFS) and OS following 2L checkpoint therapy. Several assays were carried out on patient samples, including peripheral blood immune cell phenotype and function by flow cytometry and analysis of tumour samples for immune markers using immunohistochemistry (IHC).

The study received Research Ethic Committee (REC) favourable opinion and Health Research Authority (HRA) approval on 12 October 2017 (REC ref 17/NW/0541; IRAS project 227141; clinical trial identification: IRAS No. 227141.). All patients enrolled provided written informed consent. Authors for this article adhered to Reporting Recommendations for Tumor Marker Prognostic Studies (REMARK) guidelines.^11^ Legal entity responsible for the study: The University of Manchester.

### PBMC extraction

Peripheral blood was collected in 4 × 10 ml EDTA tubes. Peripheral blood mononuclear cells (PBMCs) were extracted using SepMate™-50 tubes (STEMCELL Technologies, Cambridge, UK) and Lymphoprep (STEMCELL Technologies) according to the manufacturer’s instructions. Briefly, EDTA blood was combined and diluted 1 : 1 with wash buffer [phosphate-buffered saline (PBS; Sigma, Bournemouth, UK) supplemented with 2% fetal calf serum (Sigma)]. The diluted blood was then layered over 15 ml of Lymphoprep in multiple SepMate tubes according to the volume of blood. The tubes were then centrifuged at 800 *g* for 10 min with the brakes on. The fraction containing PBMC was then poured into fresh 50-ml falcon tubes and washed twice with wash buffer. PBMCs were counted using trypan blue frozen down at 40 × 10^6^ cells/vial and stored in liquid nitrogen until required.

### Flow cytometry analysis of PBMC phenotype and function

Briefly, PBMC vial(s) were thawed using T-cell media [RPMI (GIBCO, Renfrew, UK) supplemented 10% fetal bovine serum (Sigma), 1% Pen Strep (ThermoFisher, Warrington, UK), 30 mM HEPES (ThermoFisher), 50 μM β-mercaptoethanol (Sigma)] and then counted using trypan blue and a haemocytometer. PBMC phenotype was assessed using three antibody panels (T-cell memory/activation panel, T-cell exhaustion panel and T-cell functional panel as described in Supplementary Table S1, available at https://doi.org/10.1016/j.iotech.2024.100712). PBMCs to be assessed with the functional panel were rested for 4 h before stimulation with Dynabeads (CD3/CD28) (Invtirogen) at a 1 : 1 cell-to-bead ratio for 15 h in the presence of monensin (1 : 1000 dilution) (BioLegend, London, UK). The remaining PBMCs were rested unstimulated over 15 h. For each panel, PBMCs were surface stained with antibodies in 96-well plates at 1 million cells per well resuspended in 100 μl brilliant violet staining buffer. The cells were washed with fluorescence-activated cell sorter buffer (PBS supplemented with 2% fetal bovine serum), stained with Live/Dead green (1 : 5000 dilution) (Invitrogen, Warrington, UK), washed again with FACS buffer. The PBMC samples stained with the T-cell memory and T-cell exhaustion panels were resuspended in FACS buffer and analysed. For the PBMC samples stained with the T-cell functional panel, the cells were incubated in fixation buffer for 15 min at 4°C and washed with FACS buffer. The PBMCs were then resuspended in 1× permeabilisation buffer and incubated for 15 min at 4°C and then washed with FACS buffer. The cells were resuspended in brilliant violet staining buffer, stained for intracellular markers for 30 min at 4°C and then washed with FACS buffer before acquisition in FACS buffer using a BD LSR Fortessa.

Following the acquisition of the samples on the LSR Fortessa™, the data were analysed using Flow Jo version 10. The gating strategy for measuring T-cell subsets was based on a previously published strategy.^12^ The T stem cell memory subset was not assessed due to difficulty in identifying this subpopulation. Data were presented as immune subsets and receptor expression on defined immune subsets both in terms of percentage of expression and median fluorescence intensity.

### Evaluating immune biomarkers using immunohistochemistry (IHC) and multiplex immunofluorescence (multiplex IF)

Diagnostic biopsy samples (treatment-naïve) were evaluated by IHC and multiplex IF methods for immune biomarker cell detection from a cohort of nine patients, and in a single patient, a second sample was obtained before initiation of nivolumab therapy. A total of 12 formalin-fixed paraffin-embedded (FFPE) slides from each patient tumour block were used for histopathology analysis, including haematoxylin and eosin and one FFPE slide for each chromogenic IHC stain CD3, CD4, CD8, FOXP3, programmed death-ligand 1 (PD-L1), CD68, Rabbit IgG isotype control, Mouse IgG isotype control and three slides for multiplex IF stains with Plex A, Plex B and Plex C (Supplementary Table S2, available at https://doi.org/10.1016/j.iotech.2024.100712).

Brightfield (BF) and multiplex IF whole tissue slides were scanned at ×20 resolution on Olympus slideview VS200 and Olympus VS120 microscopes (Olympus, Life Science Solutions) in the CRUK Manchester Institute Visualisation and Irradiation Core Facility. Digital slide scans were analysed in HALO™ v3.1.1076.429 (Indica Laboratories) using adapted stock analysis algorithms (Indica Labs Multiplex IHC v2.1.1 and Indica Labs Highplex FL v.3.2.1) for respective analysis of BF and multiplex IF images. The whole slide analysis avoided regions of necrosis and glass on the digital images and only focused on tumour/stromal regions of tissue. Image classifiers grouped whole tissue slide scans into tumour and stromal areas (measured in mm^2^), giving a final readout of cell density for each of the six markers CD3, CD4, CD8, FOXP3, CD68 and PD-L1 as the number of positive cells/mm^2^.

When quantified through digital image analysis, four out of six stains (CD3, CD4, CD68 and PD-L1) showed low inter-assay variability between BF and IF tissue density scores for each stain; the inter-class correlation (ICC) ranged from 0.82 to 0.96 between the number of positive cells/mm^2^ for each stain across the 10 patient samples. CD8 and FOXP3 stains demonstrated a weaker correlation (ICC = 0.67 each). This was most likely caused by tissue-specific autofluorescence issues with CD8 staining. FOXP3 staining appeared granular with some large cells in 3 out of 10 patient samples, indicating tumour cells and not immune cells being stained for FOXP3-positive T-regulatory marker. Taken together, the chromogenic assay was considered the gold standard and used for downstream analyses. Assessment of PD-L1 tumour proportion score was carried out manually based on pathologist measurement of PD-L1 slides stained with the Ventana PD-L1 (SP263) assay that is used routinely in NHS histopathology laboratories.

### Statistical analysis

Progression-free survival 1 (PFS1) was defined as the time from the first dose of 1L TKI (sunitinib or pazopanib) to the earliest date of disease progression or death, and PFS2 from the time of the first dose of nivolumab to disease progression or death. PFS in the 2L setting (PFS2) was compared to the duration of therapy in the 1L setting (DOT1), and the logarithm of this ratio (PFS2/DOT1) was reported graphically. Clinical benefit rate (CBR) was defined as patients having complete response or partial response (PR) or stable disease (SD) for at least 6 months. Patients who achieved CBR were considered ‘responders’. Immune-related adverse effects (irAEs) were identified through Common Terminology Criteria for Adverse Events version 4.1 and assessed through a follow-up period on nivolumab. The clinically relevant irAEs were those of grade 3-4 or requiring a course of steroids. CBR, irAEs, PFS and OS on nivolumab were used as the clinical outcome endpoints for biomarker analysis of peripheral blood-based flow cytometry data. Derived categorical variables were created using the median of the percentages of cells in the baseline samples (>median = high, <median = low). The primary endpoint was to determine the association between specific T-cell subsets with clinical outcomes tested using Wilcoxon rank sum for the CBR and dichotomised percentage of expression of immune cell subsets. The Benjamin–Hochberg method was used for correction for multiple hypothesis testing, and *q* value < 0.005 was considered significant.

The log-rank test was used to assess significant differences in OS and PFS. Immune cell subsets associated with significant patient outcomes by log-rank were analysed by Cox regression analysis to quantify the hazard ratio (HR). The significance level for these analyses was set at *P* value < 0.05. STATA 16 software (Stata Statistical Software: Release 16. College Station, TX: StataCorp LLC) was used to run our analysis.

## Results

### Baseline characteristics of patients

Twenty patients, 13 males and 7 females, were recruited to receive 2L nivolumab after progression on the 1L anti-VEGF TKI. The median age was 64 years (range 36-83 years). The majority had clear cell histology (*n* = 17) (Table 1). The median time of follow-up from the time of consent was 12 months (range 0.4-44.33 months). All but one patient received at least one dose of nivolumab. One patient succumbed to his disease before C1D1. Five patients had immune-related adverse events. The median OS from the time of starting 1L TKI was 31.2 months [interquartile range (IQR) 18.6-47 months], and from the time of starting nivolumab was 12 months (IQR 6.1-23.9 months).

**Table 1:** Baseline characteristics of the included patients

Characteristic	Number of patients, *n* = 20 (%)
Gender	
Male	13 (65)
Female	7 (35)
Median age (years)	64 (36-83)
Radical nephrectomy	
Yes	12 (60)
No	8 (40)
Histology	
Clear cell (8310/3)^a^	17 (85)
Mixed (8959/0)^a^	3 (15)
Race	
White	19 (95)
Asian	1 (5)
First-line treatment	
Sunitinib	11 (55)
Pazopanib	9 (45)
Distant metastasis	
Lung	13 (65)
Bone	8 (40)
Lymph nodes	7 (35)
Pancreas	3 (15)
Stage at diagnosis	
Stage 1	1 (5)
Stage 2	4 (20)
Stage 3	6 (30)
Stage 4	9 (45)

ICD, International Classification of Diseases.

a According to ICD code.

### Clinical outcomes

**Figure 1:**
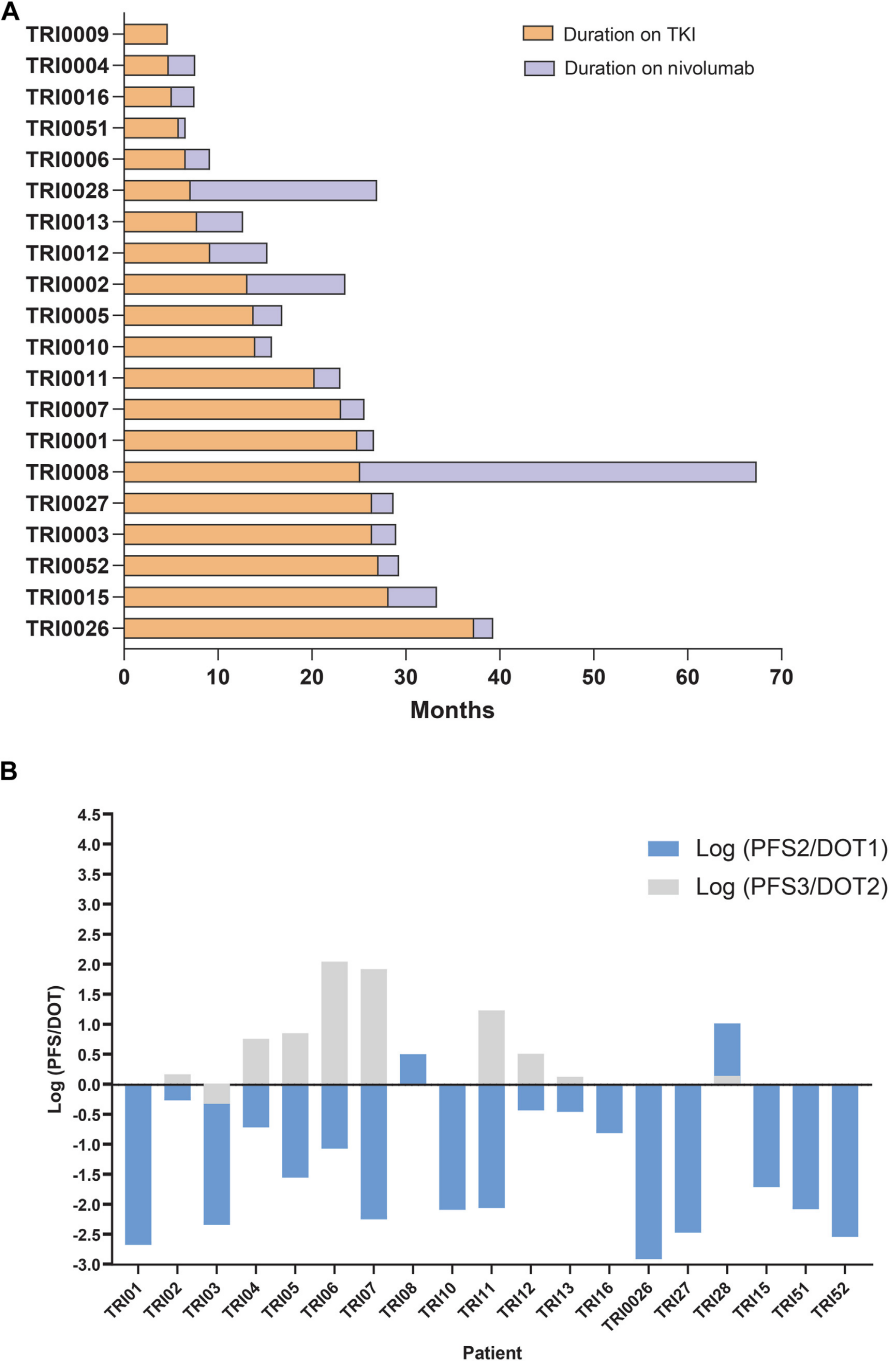
**Clinical outcomes on 1L TKI and 2L nivolumab.** (A) Swimmer’s plot indicates the duration on TKI and nivolumab. (B) The logarithm ratio of PFS to the DOT for the first and second lines. Log (PFS/DOT) > 0 indicates a relative superior benefit. 1L, first-line; 2L, second-line; DOT, duration of treatment; PFS, progression-free survival; TKI, tyrosine kinase inhibitor.

The study met its primary endpoint in identifying a significant association between immune cell subsets and the clinical outcomes to nivolumab. The median PFS on 1L TKI was 13.9 months (IQR 6.8-25.9 months). No significant difference was observed in the response rate (*P* = 0.472) or PFS (*P* = 0.68) in sunitinib versus pazopanib subgroups. The median PFS on nivolumab was 2.57 months (IQR 2.2-5.1 months). Four patients achieved a CBR at 6 months: two PRs and two SDs. Responders to nivolumab were associated with longer median OS (median = 26.8 months) when compared to the non-responders (median OS = 10 months) with HR 0.24; 95% CI 0.53-1.08; *P* = 0.06, and significantly longer PFS (median = 15.1 months) when compared to the non-responders (median PFS = 2.5 months); HR 0.10; 95% CI 0.01-0.42; *P* < 0.05. Durations of response on 1L TKI and 2L nivolumab are illustrated in Figure 1A. The increase in neutrophils/lymphocytes (N/L) ratio at 6 weeks did not predict worse PFS to nivolumab (HR 1.18; 95% CI 0.41-3.36; *P* = 0.756).

**Figure 2:**
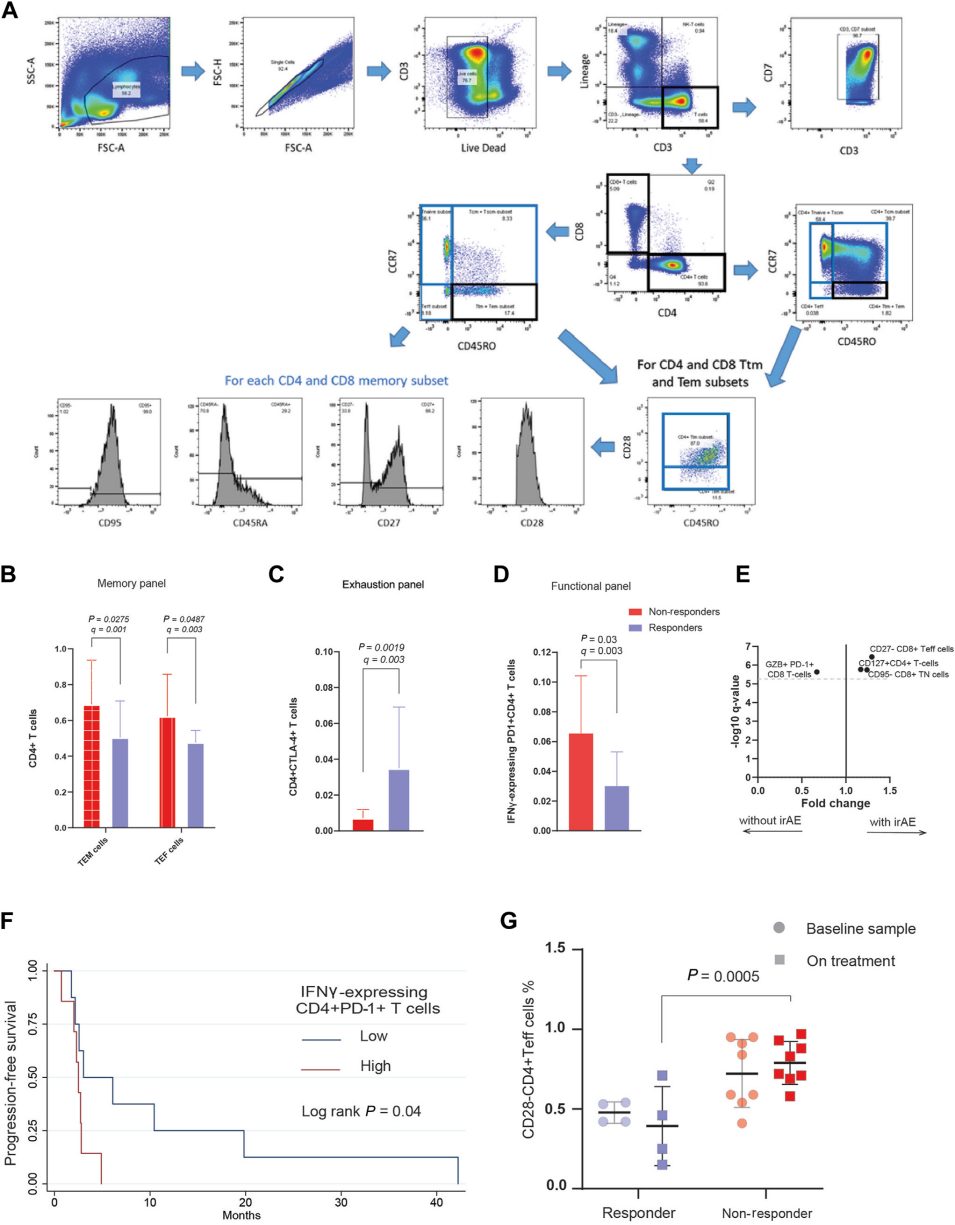
**Peripheral blood immune cell subpopulations and response to nivolumab.** (A) Gating strategy for T cells in the memory panel. Barplots for the mean expression fraction of immune cell subsets in the memory (B), exhaustion (C) and functional panels (D) according to clinical response status; whiskers represent the standard deviation, x-axis is the cell fraction. (E) Plot of significant cells enriched in patients who developed irAEs; dashed line is at *q* = 0.005. (F) Kaplan–Meier curve showing patients with low expression fraction had better progression-free survival (G). Box plot showing responders had a significant reduction in CD4+T_EF_ cell fraction compared to non-responders at 3 months on nivolumab. *P* value calculated using two-way ANOVA test, clinical responders coloured with fainter colour. ANOVA, analysis of variance; CBR, clinical benefit rate; IFN, interferon; irAEs, immune-related adverse events; OS, overall survival; PD-1, programmed cell death protein 1; PFS, progression-free survival.

We applied the logarithm ratio of PFS to DOT of the prior line of treatment to estimate the relative benefit of the subsequent line (see the Materials and Methods section). Only 2/19 had Log (PFS2/DOT1) > 0, indicating a minority of patients had a relatively superior outcome with nivolumab as compared to 1L TKI. Meanwhile, out of the 10 patients who received a third-line TKI, 9 had Log (PFS3/DOT2) > 0, indicating a majority of patients had relative superior outcomes with third-line TKI as compared to 2L nivolumab (Figure 2B).

### Peripheral blood immune cell subpopulations and response to nivolumab

Baseline samples were analysed for 20 patients, of which 12 also had a serial sample collected at 3 months’ follow-up. We developed three panels and applied gating strategies for measuring T-cell subsets in PBMC isolated from peripheral blood samples, as illustrated in Figure 2A and Supplementary Figures S2 and S3, available at https://doi.org/10.1016/j.iotech.2024.100712.

We examined the association of specific T-cell subsets and clinical outcomes and immune-related toxicities (Figure 2B-F). Five patients (25%) in our study developed irAEs. Using the T-cell memory antibody panel, the mean fraction of CD45RA+CD4 T_EM_ and CD28+CD4+ T_EF_ cells were significantly higher in non-responders compared to responders (0.70 versus 0.50, *q* = 0.001 and 0.62 versus 0.47 *q* = 0.003, respectively) (Figure 2B). In the T-cell exhaustion panel, the cytotoxic T-lymphocyte associated protein 4+ CD4+ T cells were significantly enriched in responders compared to non-responders (0.04 versus 0.01, *q* = 0.003) (Figure 2C). Using the functional T-cell antibody panel, the fraction of CD4+ T cells and CD8+ T cells expressing granzyme B and the cytokines interleukin-2, tumour necrosis factor-α and interferon (IFN)-γ in response to *ex vivo* stimulation using Dynabeads (CD3/CD28) were assessed. Responders had a significantly low expression fraction of IFN-γ-expressing PD-1+ CD4+ T cells compared to non-responders (0.3 versus 0.6, *q* = 0.003) (Figure 2D). In addition, patients with low levels of IFN-γ-expressing programmed cell death protein 1+ CD4+ T cells were associated with better PFS compared to patients with high levels (HR 0.30, 95% CI 0.10-1.00, log-rank *P* = 0.04) (Figure 2F).

Across the three antibody panels, we identified three T-cell subsets that were associated with higher likelihoods of developing irAEs, including CD8-naïve and effector T cells and CD127+ CD4+ T cells with fold change of 1.24, 1.29 and 1.17 (*q* < 0.005), respectively (Figure 2E).

We examined the changes of peripheral blood T-cell subsets from the baseline to 3-month timepoints according to the clinical response status. CD28-CD4+Teff cells showed a significant reduction in responders compared to non-responders (0.4 versus 0.8 two-way analysis of variance *P* = 0.0005) (Figure 2G).

### Tumour-associated immune cell densities

**Figure 3:**
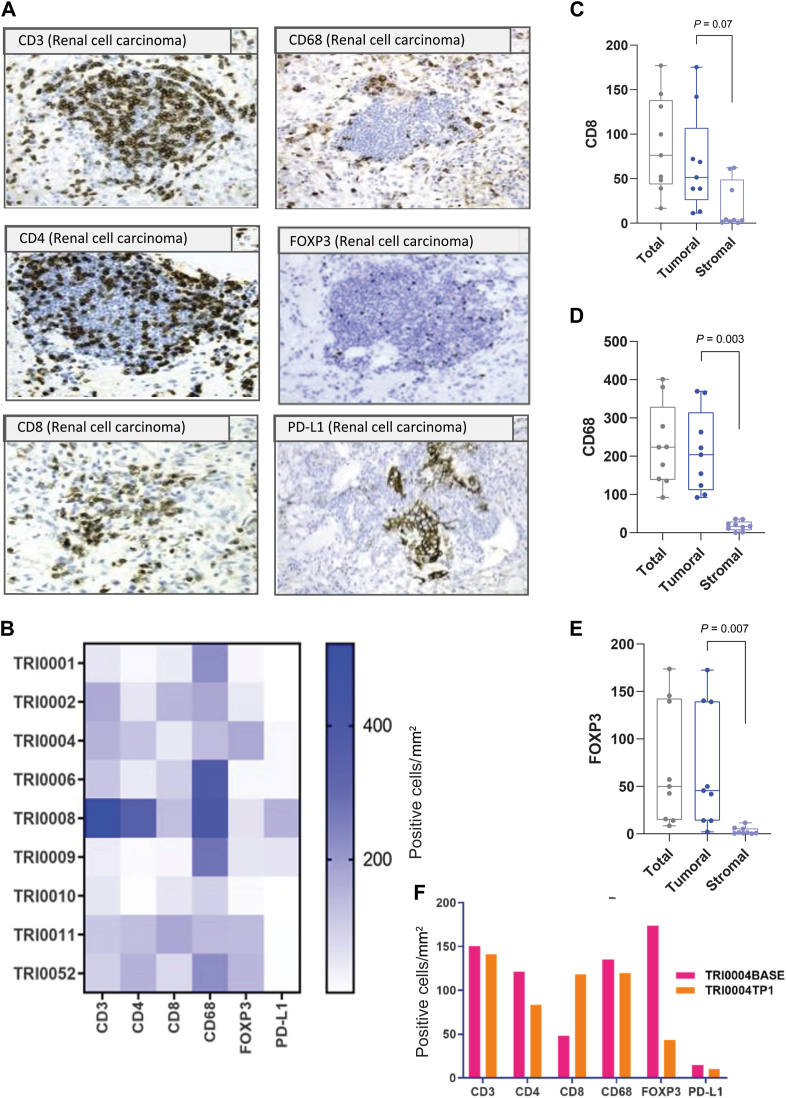
**Tumour-associated immune cell densities.** (A) Example images of six chromogenic immune biomarkers in renal cell carcinoma FFPE tissue. (B) Heatmap of the positive cells/mm^2^ expressing the six immune biomarkers per individual patient. Boxplots of the immune cell densities measured as positive cells/mm^2^ that express CD8 (C), CD68 (D) and FOXP3 (E) in tumoral and stromal compartments. (F) The change in immune cell densities in one patient with two serial samples, one taken at baseline and another collected post-treatment with TKI. FFPE, formalin-fixed paraffin-embedded; PD-L1, programmed death-ligand 1; TKI, tyrosine kinase inhibitor.

Using IHC and image analysis approaches, we evaluated the immune cell densities of FFPE diagnostic tumour samples from nine patients. The T-cell subpopulations were significantly enriched in tumoral compared to stromal regions, including CD8+ T cells, FOXP3+ T regs and CD68+ macrophages (*P* < 0.05) (Figure 3C-E). The median immune cell densities were 113 positive cells/mm^2^ for CD3, 51 for CD4, 223 for CD68 and 76 for CD8. No statistically significant differences were found in the relative values of the six immune-oncology biomarkers (CD3, CD4, CD8, CD68, FOXP3 and PD-L1) among the nine RCC samples, as shown in (Figure 3B). However, it is worth noting that TRI0008 exhibited a higher number of CD3-, CD4-, CD8- and CD68-positive cells/mm^2^ compared to the other eight samples. Patient TRI0008 was the only patient in this cohort to experience a durable clinical response to the TKI inhibitor and a sequential nivolumab treatment (Figure 1).

Univariate Cox regression analyses could not identify a significant association between immune cell densities and PFS to 1L TKI (*P* < 0.05). Meanwhile, immune cell densities were significantly higher in the two patients who achieved CBR to nivolumab compared to the seven patients without CBR (mean CD3+ cells density 346 versus 87 cells/mm^2^, rank sum *P* = 0.04) (Supplementary Table S3, available at https://doi.org/10.1016/j.iotech.2024.100712). Patients with favourable immune cell profile (high CD8 and low FOXP3 density) were associated with longer PFS to nivolumab (log-rank *P* = 0.035, adjusted for gender and ECOG PS). In one patient with baseline and post-TKI biopsy, we could show that TKI led to an increase in CD8+ T cells and a decrease in CD4+ FOXP3+ T regs (Figure 3F).

## Discussion

The primary objective of this study was to evaluate immunological signatures in mRCC patients following disease progression on 1L TKI therapy with either pazopanib or sunitinib. We conducted exploratory biomarker analyses and reported several insightful findings using flow cytometry and IHC.

Firstly, we examined the relative benefit of 2L nivolumab compared to 1L and 3L TKI. We found that a minority of patients had relatively superior outcomes with nivolumab when used after 1L TKI. Meanwhile, the majority of patients had superior outcomes with third-line TKI when used after nivolumab. These observations suggest that patients who stopped responding to upfront TKI can still benefit from different TKIs if used in a later line of treatment.^13^ However, this notion requires further investigation due to the limited number of patients who received a third-line TKI in our cohort.

Secondly, our results suggested certain T-cell subsets at baseline samples predicted clinical outcomes for nivolumab treatment in patients with mRCC. We identified subsets of peripheral CD4 T cells, including effector and memory effector cells that conferred resistance to nivolumab and were significantly expanded in non-responders. The predictive capacity of peripheral CD4 T-cell subsets was shown previously in patients with non-small-cell lung cancer^14^ and melanoma^15^ receiving nivolumab.

Our results found that irAEs were highly associated with CD127+ expressed on CD4+ T cells. This can be explained by the association between CD127+ and many immune-related diseases, e.g. multiple sclerosis and skin reaction to drugs. Blockade of its pathway suggested for autoimmune disease treatment.^16^^,^^17^ Of note, the two patients with the highest CD127+ CD4+ T-cell percentages developed immune-related colitis.

Immune cell densities were significantly higher in patients who achieved CBR to nivolumab compared to patients without CBR. High CD8 and low FOXP3 are associated with longer PFS to nivolumab. Our findings highlight the importance of investigating the immune modulatory effects of TKI along with immune checkpoint therapy.^18^ The combination of TKIs with immune checkpoint therapy offers a dual mechanism of action, targeting cancer cells and the tumour microenvironment, potentially enhancing treatment efficacy and overcoming resistance mechanisms. Further exploration of TKI-induced immune modulation shows promise for refining therapeutic approaches and enhancing outcomes in cancer immunotherapy.

One of the limitations of our study is the small sample size. In addition, we did not analyse serum cytokines. A prior report showed that pazopanib induced changes in the serum cytokine profile.^19^ However, our study characterised key immune cells in peripheral blood that were associated with predictive capacity. Further validation using transcriptomic analysis to characterise the gene expression profile of identified cells and their predictive capacity is needed.^20^

In conclusion, monitoring peripheral blood immune cell subsets may be of value in predicting outcomes and adverse events of anti-VEGF TKI and nivolumab. Future research will apply methods developed for this study to larger numbers of patients in combination settings to gain a greater understanding of the roles of particular T-cell subsets in predicting response and toxicities.
