# Supplementary material for: Immune biomarker evaluation of sequential tyrosine kinase inhibitor and nivolumab monotherapies in renal cell carcinoma: the phase I TRIBE trial
Source: Immunooncol Technol. 2024 Mar 18;22:100712. doi: 10.1016/j.iotech.2024.100712 (PMC11059457; doi:10.1016/j.iotech.2024.100712)
Supplement: Supplementary data [file mmc1.docx]

**Supplementary Information File**

**Immune biomarker evaluation of sequential tyrosine kinase inhibitor and nivolumab monotherapies in renal cell carcinoma: The phase I TRIBE trial**

**Shohdy KS et al.**

**Supplementary Figures:**

Supplementary Figure 1. The TRIBE trial design with inclusion criteria and the sample collection process.

**Supplementary Figure 2.** Gating strategy for the T cell exhaustion flow cytometry antibody panel.

**Supplementary Figure 3.** Gating strategy for the T cell functional flow cytometry antibody panel.

**Supplementary Tables:**

Supplementary Table 1. List of antibodies used in the flow cytometry panels.

**Supplementary Table 2.** Primary anti-human antibodies used for chromogenic and Immunofluorescence IHC.

**Supplementary Table 3.** Immune cell densities across the patients.


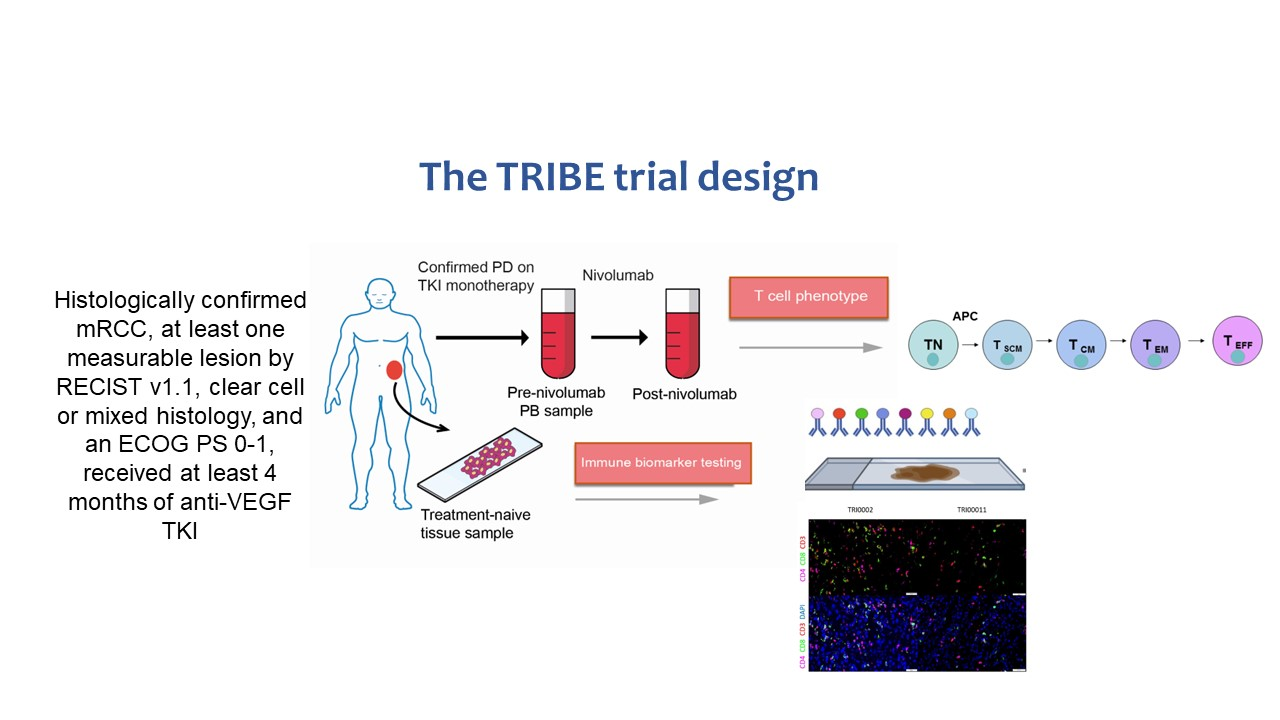


Supplementary Figure 1 The TRIBE trial design with inclusion criteria and the sample collection process.

**
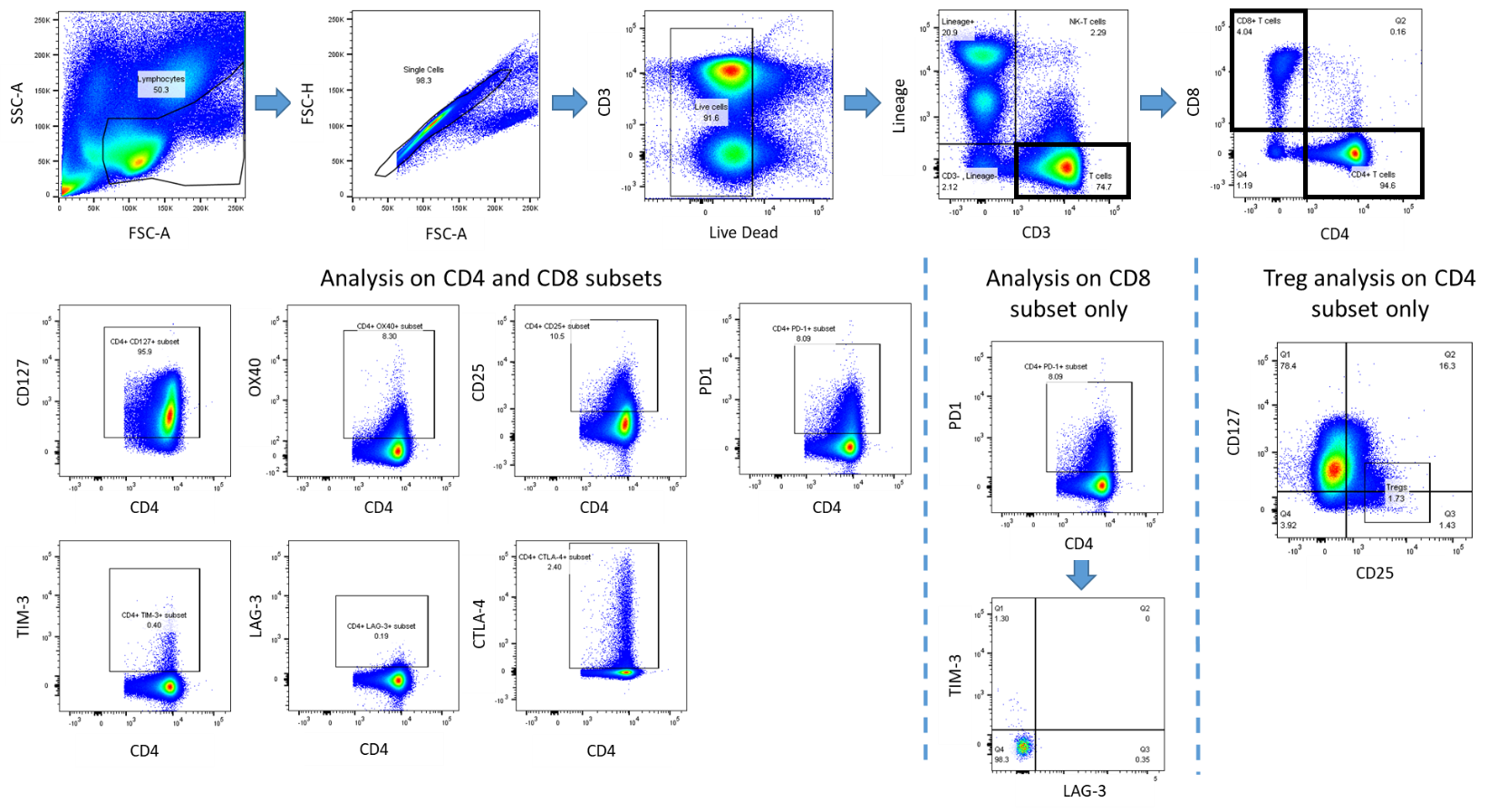
**

**Supplementary Figure 2.** Gating strategy for the T cell exhaustion flow cytometry antibody panel.

**
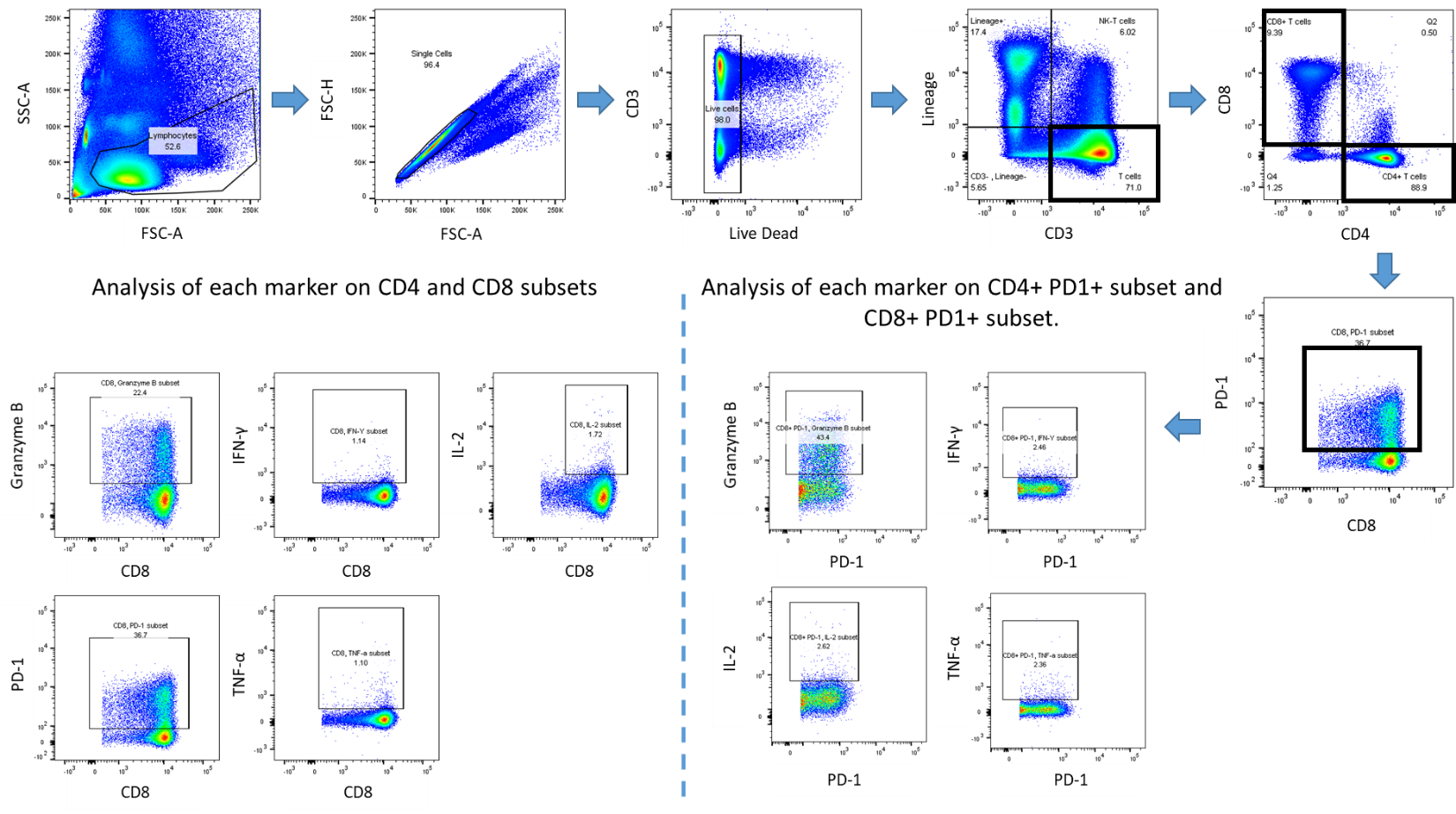
**

**Supplementary Figure 3.** Gating strategy for the T cell functional flow cytometry antibody panel.

Supplementary Table 1. List of antibodies used in the flow cytometry panels.

| **Antibody** | **Company** | **Catalogue No.** | **Panel** |
| --- | --- | --- | --- |
| CD3 PerCP/Cy5.5 | BioLegend | 317336 | Present in all panels |
| CD4 BV785 | BioLegend | 317442 |  |
| CD8 APC-H7 | BD Biosciences | 560179 |  |
| CD56 Pe Cy7 | BioLegend | 318318 |  |
| CD14 PeCy7 | BioLegend | 325618 |  |
| CD20 Pe Cy7 | BioLegend | 302312 |  |
| CD45RA BV605 | BioLegend | 304134 | Present in the memory/activation panel |
| CD45RO BV421 | BioLegend | 304224 |  |
| CCR7 APC | BioLegend | 353214 |  |
| CD95 BV 711 | BioLegend | 305644 |  |
| CD28 PE/dazzle 594 | BioLegend | 302942 |  |
| CD27 BV510 | BioLegend | 356420 |  |
| CD7 PE | BioLegend | 343106 |  |
| CD25 BV711 | BioLegend | 302636 | Present in the exhaustion panel |
| CD127 BV421 | BioLegend | 351310 |  |
| PD-1 PE | BioLegend | 329906 |  |
| TIM-3 Pe/Dazzle 594 | BioLegend | 345034 |  |
| OptiBuild™ CD134 (OX40) BV510 | BD Biosciences | 745040 |  |
| LAG-3 AF647 | BioLegend | 369304 |  |
| CTLA-4 PE | BioLegend | 369604 |  |
| IL-2 BV421 | BioLegend | 500328 | Present in the functional panel |
| Granzyme B BV510 | BioLegend | 563388 |  |
| TNF-α BV650 | BioLegend | 502938 |  |
| IFN-γ BV711 | BioLegend | 502540 |  |
| Il-10 APC | BioLegend | 506807 |  |
| PD-1 BV605 | BioLegend | 329924 |  |
| Live/dead AF 488 | ThermoFisher Scientific | L-34961 | Present in all panels |

**Supplementary Table 2.** Primary anti-human antibodies used for chromogenic and immunofluorescence IHC.

| **Marker** | **Clone** | **Species** | **Isotype** | **Cellular**  **localisation** | **Antibody**  **source** | **Dilution**  **Brightfield** | **Dilution**  **IF** |
| --- | --- | --- | --- | --- | --- | --- | --- |
| **CD3** | NCL-L-CD3- 565 | Ms monoclonal | IgG1 | Nucleus | Leica Biosystems | 1:150 | 1:150 |
| **CD4** | CD4-368-L- CE | Ms. Monoclonal | IgG1,  kappa | Membrane | Leica Biosystems | 1:50 | 1:50 |
| **CD8** | C8-144B | Ms.  Monoclonal | IgG1,  kappa | Membrane | Cell Marque | 1:50 | 1:100 |
| **CD68** | M087601-2 | Ms. Monoclonal | IgG3,  kappa | Membrane | Dako | 1:250 | 1:500 |
| **FOXP3** | ab20034 | Ms.  Monoclonal | IgG1 | Nucleus | Abcam | 1:75 | 1:200 |
| **PD-L1** | E1L3N | Rb. monoclonal | Rb IgG | Membrane and cytoplasm | Cell Signalling Technology | 1:400 | 1:1000 |

| **Supplementary Table 3.** Immune cell densities across the patient samples. | | | |
| --- | --- | --- | --- |
| **FFPE Slide** | **Total TILs (Cells/mm2)** | **Epithelial TILs (Cells/mm2)** | **Stromal TILs (Cells/mm2)** |
| TRI0001_CD3 | 51.3 | 51.3 | 0 |
| TRI0001_CD4 | 15.1 | 14.9 | 0.1 |
| TRI0001_CD68 | 223.4 | 221.9 | 1.6 |
| TRI0001_CD8 | 39 | 38.8 | 0.3 |
| TRI0001_FOXP3 | 15.5 | 14 | 1.4 |
| TRI0001_PDL1 | 1.7 | 1.7 | 0 |
| TRI0002_CD3 | 169.7 | 104.1 | 65.6 |
| TRI0002_CD4 | 51 | 50.8 | 0.1 |
| TRI0002_CD68 | 177.6 | 154.2 | 23.5 |
| TRI0002_CD8 | 145.3 | 142.1 | 3.2 |
| TRI0002_FOXP3 | 42.6 | 41.9 | 0.7 |
| TRI0002_PDL1 | 1.1 | 1 | 0.1 |
| TRI0004BASE_CD3 | 150.6 | 29.3 | 121.2 |
| TRI0004BASE_CD4 | 120.9 | 70.6 | 50.4 |
| TRI0004BASE_CD68 | 135.5 | 99.4 | 36.1 |
| TRI0004BASE_CD8 | 48.2 | 11.2 | 37 |
| TRI0004BASE_FOXP3 | 173.8 | 172.6 | 1.3 |
| TRI0004BASE_PDL1 | 15 | 13.1 | 1.9 |
| TRI0004TP1_CD3 | 141.5 | 105.3 | 36.2 |
| TRI0004TP1_CD4 | 83.7 | 45.4 | 38.3 |
| TRI0004TP1_CD68 | 119.6 | 25.7 | 93.9 |
| TRI0004TP1_CD8 | 118 | 95.2 | 22.8 |
| TRI0004TP1_FOXP3 | 43.1 | 33.7 | 9.4 |
| TRI0004TP1_PDL1 | 10.1 | 3.6 | 6.5 |
| TRI0006_CD3 | 114.8 | 108.9 | 5.9 |
| TRI0006_CD4 | 40.8 | 31.4 | 9.4 |
| TRI0006_CD68 | 380.7 | 369.7 | 11 |
| TRI0006_CD8 | 99.7 | 38.7 | 61 |
| TRI0006_FOXP3 | 14 | 13.9 | 0.1 |
| TRI0006_PDL1 | 14.8 | 14.8 | 0 |
| TRI0008_CD3 | 522.8 | 215.9 | 306.9 |
| TRI0008_CD4 | 354.2 | 162.6 | 191.6 |
| TRI0008_CD68 | 401.1 | 366.1 | 35 |
| TRI0008_CD8 | 131.1 | 68.8 | 62.3 |
| TRI0008_FOXP3 | 57.2 | 45.5 | 11.6 |
| TRI0008_PDL1 | 155.4 | 154.2 | 1.2 |
| TRI0009_CD3 | 37.2 | 27 | 10.2 |
| TRI0009_CD4 | 11.2 | 9.8 | 1.3 |
| TRI0009_CD68 | 278 | 263.2 | 14.8 |
| TRI0009_CD8 | 16.8 | 13.1 | 3.6 |
| TRI0009_FOXP3 | 49.9 | 49.5 | 0.5 |
| TRI0009_PDL1 | 52.7 | 52.7 | 0 |
| TRI00010_CD3 | 48.4 | 36.1 | 12.3 |
| TRI00010_CD4 | 7.2 | 6.2 | 1 |
| TRI00010_CD68 | 92.1 | 92.1 | 0.1 |
| TRI00010_CD8 | 52.2 | 51.4 | 0.8 |
| TRI00010_FOXP3 | 8.4 | 2.1 | 6.3 |
| TRI00010_PDL1 | 1.4 | 1 | 0.4 |
| TRI00011_CD3 | 113.2 | 108 | 5.3 |
| TRI00011_CD4 | 131.7 | 121.7 | 10 |
| TRI00011_CD68 | 140.6 | 123.6 | 17 |
| TRI00011_CD8 | 177.1 | 175.2 | 2 |
| TRI00011_FOXP3 | 139.5 | 139.1 | 0.4 |
| TRI00011_PDL1 | 3.4 | 3.1 | 0.3 |
| TRI0052_CD3 | 96.2 | 79.9 | 16.3 |
| TRI0052_CD4 | 156.3 | 133.5 | 22.9 |
| TRI0052_CD68 | 224.4 | 203.8 | 20.6 |
| TRI0052_CD8 | 76.2 | 72 | 4.2 |
| TRI0052_FOXP3 | 145.5 | 140.2 | 5.3 |
| TRI0052_PDL1 | 3.6 | 3.3 | 0.3 |
